# Client involvement in motivational interviewing sessions targeting substance abuse treatment in the criminal justice system

**DOI:** 10.1186/1940-0640-10-S1-A67

**Published:** 2015-02-20

**Authors:** Teneshia Thurman, Faye S Taxman, Scott T Walters

**Affiliations:** 1 Criminology, Law & Society, George Mason University, Fairfax, VA, 22030, USA; 2 Behavioral and Community Health, University of North Texas Health Science Center, Fort Worth, TX, 76107, USA

## Background

Motivational interviewing (MI) is a counseling technique used to elicit change by exploring an individual’s desire and commitment to making changes. In this study, probation clients are randomized to a two-session MI intervention occurring approximately 3–4 weeks apart. The first session primarily focuses on building motivation to change substance use, engage in treatment, and make other changes to improve probation outcomes; the second session focuses on goal setting and social support.

## Methods

This paper examines responses from 96 clients who completed the first counselor-driven MI session. Researchers extracted client reasons associated with commitment to probation and treatment. Analyses matched the clients’ responses to the MI session with quantitative data on motivation to change, demographics, and criminal history obtained during the baseline assessment.

## Results

Clients generally reported higher levels of treatment motivation during the MI session as compared to the scales from the CJ Client Evaluation of Self and Treatment (CEST). Results from the CEST indicated that the majority of the sample (79.2%) had low/moderate levels of motivation to seek help and recognize that they have a problem (82.3%). However, on a scale of 1–10, clients reported high commitment to complete probation (M = 9.67) and treatment/decreasing use (M = 8.30). Individuals under 36 years old who scored low/moderate on a criminal justice (CJ) risk tool, and males indicating that they did not want to go to jail reported higher commitment ratings. During the MI session, the primary reasons for increased motivation were family, tired of being in the CJ system, not wanting to go back to jail, and tired of using. For female probationers, family was the most frequently stated reason (28.6%) for being committed to finishing probation. Employment was the primary reason given for wanting to change substance use behavior, particularly among probationers under 36 years old. Older individuals were more likely to be tired of using drugs. During the MI session, clients’ goals for the next 30 days mostly dealt with probation issues like attending the first probation appointment (53.1%). Treatment-related goals (e.g., making a treatment provider appointment (26%) were less frequent.

## Conclusion

An MI counseling session appears to be drawing out greater intrinsic reasons and commitment for making changes in probation, treatment, and other behaviors. These behaviors, in turn, may reduce the subsequent risk of criminality. Knowing what motivates individuals to change their behavior can help practitioners to focus on those factors that are most likely to facilitate behavior change.

## Trial registration

NCT01891656

